# Chemical Profile Characterization of Fruit and Vegetable Juices after Fermentation with Probiotic Strains

**DOI:** 10.3390/foods13071136

**Published:** 2024-04-08

**Authors:** Ioanna Mantzourani, Anastasios Nikolaou, Yiannis Kourkoutas, Athanasios Alexopoulos, Marilena Dasenaki, Artemis Mastrotheodoraki, Charalampos Proestos, Nikolaos Thomaidis, Stavros Plessas

**Affiliations:** 1 Laboratory of Food Processing, Faculty of Agriculture Development, Democritus University of Thrace, 68200 Orestiada, Greece; anikol@mbg.duth.gr (A.N.); splessas@agro.duth.gr (S.P.); 2 Laboratory of Applied Microbiology & Biotechnology, Department of Molecular Biology & Genetics, Democritus University of Thrace, 68100 Alexandroupolis, Greece; ikourkou@mbg.duth.gr; 3 Laboratory of Microbiology, Biotechnology & Hygiene, Faculty of Agriculture Development, Democritus University of Thrace, 68200 Orestiada, Greece; alexopo@agro.duth.gr; 4 Laboratory of Food Chemistry, Department of Chemistry, National and Kapodistrian University of Athens, Panepistimiopolis Zografou, 15771 Athens, Greece; mdasenaki@chem.uoa.gr (M.D.); artemismastr@chem.uoa.gr (A.M.); harpro@chem.uoa.gr (C.P.); 5 Laboratory of Analytical Chemistry, Department of Chemistry, National and Kapodistrian University of Athens, Panepistimiopolis Zografou, 15771 Athens, Greece; ntho@chem.uoa.gr

**Keywords:** fruit juices, fermentation, LAB, antioxidant activity, phenolic profile

## Abstract

Lactic acid bacteria (LAB) are widely applied for fermentation purposes in dairy and non-dairy food matrices with beneficial technological and health-promoting properties. This study describes the effect of two lactic acid bacteria, namely, *Lactiplantibacillus paracasei* SP5 and *Pediococcus pentosaceus* SP2, on the phenolic profiles, antioxidant activities, total phenolic content (TPC), carotenoid content, and sensorial profile of two different mixed fruit juices. After 48 h of fermentation, both LABs retained viability over 9 Log CFU/mL in both juices. The TPC, zeaxanthin + lutein, β-carotene content, and antioxidant activity (AA) were elevated for both LABs and mixed juices after 48 h of fermentation compared to control samples. Regarding the phenolic profile, both juices exhibited a significant decrease in chlorogenic acid levels, while quinic acid and tyrosol concentrations showed notable increases.

## 1. Introduction

Currently, there is a great interest in the food industry and research into producing fruit and vegetable juices with novel technological properties and advanced health benefits for consumers’ health. In this vein, mixed fruit juices may be used as fermentation substrates with probiotic strains, providing beverages with high nutritional value and health-promoting abilities. More specifically, lactic acid bacteria (LAB) have been applied for fermentation to plant-based substrates producing food products with advanced antioxidant activity, antimicrobial properties, and acceptable sensorial evaluation [1].

Fruits like oranges, apples, grapes, pomegranates, and their juices are consumed in large amounts worldwide every day. Their consumption has become even greater after consumers’ awareness about fruits’ nutritional value and health benefits. These juices are rich in polyphenols, such as isoflavones, flavonoids, and phenolic acids [2].

Additionally, red fruits like strawberries, black chokeberries, bilberries, blood oranges, cherries, and sour cherries are sources of anthocyanins, flavonoids, and phenolic acids [3]. These bioactive compounds undergo antioxidant, anticancer, and cardiovascular protective properties [4].

It has been established that the fermentation of fruit juices using LAB has been shown to improve the functional properties of the substrate [5]. Emerging research has proved that the biotransformation and the bioavailability of phenolic compounds during LAB fermentation were elevated [6]. Furthermore, other researchers’ findings have indicated that the antioxidant activity, total phenolic content, and carotenoid content in fermented juices are usually increased after fermentation [7].

Various other lactic acid bacteria (LAB) have been applied to other fruit and vegetable juices for fermentation; likewise, *Lactiplantibacillus paracasei* SP5 has also been examined for its technological properties to apple–orange–carrot juice [8]. On the other hand, other LAB are being examined in this kind of fermentation. *Pediococcus pentosaceus* SP2 has only been applied for the production of sourdough bread [9] and has never been examined for its ability to ferment fruit juices before. We hypothesized that the fermentation process would enhance the nutritional value of the juice samples.

In the present study, two potential probiotic strains, *Lactiplantibacillus paracasei* SP5 and *Pediococcus pentosaceus* SP2, were applied for 24 h and 48 h of fermentation using two different popular commercial fruit juices. The first one was a mixture of four fruits, and the second one contained nine red fruits and one vegetable. The viability of the two lactobacilli strains during fermentation was determined, and the concentration of total sugars and organic acids was measured pre- and post-fermentation. Additionally, carotenoid profiling (zeaxanthin + lutein, β-carotene, lycopene) and comprehensive phenolic profiling were performed using HPLC-DAD and LC-QToF/MS analysis, respectively. The TPC and the antioxidant activity of the fermented and the unfermented juice samples were determined as well.

## 2. Materials and Methods

### 2.1. Fruit and Vegetable Juice Preparation

Both juices examined were commercially available in Greece under the brand Olympos (Larissa, Greece). The 4-fruit juice contained red apple (52%), orange (4.5%), pomegranate (27.1%), and red grape (16.4%) from concentrated juices, and the 9-fruit juice included red apple, red grape, cherry, pomegranate, sour cherry, strawberry, black chokeberry, blueberry, blood orange, and black carrot (100%) from concentrated juices as well.

### 2.2. Juice Fermentation

Initially, the juice was divided into quantities of 100 mL inside Erlenmeyer flasks and was pasteurized at 80 °C for 15 min [10]. The pasteurized juices without the addition of probiotic strains were used as controls. Subsequently, 1 g of wet biomass *L. paracasei* SP5 and *P. pentosaceus* SP2 was added to each flask (in triplicate) and left to ferment for 24 h and 48 h at 30 °C. The cell density was 10^10^ CFU/mL.

### 2.3. Microbiological Analysis of Juices

Microorganisms responsible for the spoilage of the juice, mostly yeasts and molds, were detected during fermentation. In addition, the microbial counts of lactic acid bacteria (LAB) were also screened in triplicate before and after 24 h and 48 h of fermentation. Therefore, a representative amount of 10 mL from each juice sample was blended with 90 mL of sterilized 1/4 Ringer’s solution (Sigma-Aldrich, St. Louis, MO, USA) and subjected to serial dilutions.

The following tests were performed: (i) lactobacilli [Gram (+), catalase (−)] on acidified MRS agar (Oxoid Ltd., Hampshire, UK) at 37 °C for 48 h anaerobically (Anaerobic jar, Anerocult C, Merck, Rahway, NJ, USA); (ii) yeasts and molds on malt agar (Oxoid Ltd., Hampshire, UK) (pH was adjusted to 4.5 using a sterile solution of 10% lactic acid) at 30 °C for 48 h. All incubations were further extended up to 120 h; however, no extra colonies were observed. Gram staining and catalase tests were performed for LAB confirmation. Results are presented as a log of mean colony-forming units (CFU) per mL of each juice.

### 2.4. Determination of Total Phenolic Content (TPC)

The total phenolic content of each juice sample was determined according to the Folin–Ciocâlteu method. Moreover, 20 μL of the sample was placed into a cuvette with 1.58 mL of water and 100 μL of Folin–Ciocâlteu reagent and was mixed well. After 5 min, 300 μL of aqueous sodium carbonate solution (20% *w*/*v*) was added to adjust the pH of the reagent. All solutions were kept at 20 °C for 2 h and then their absorbance was measured at 765 nm using a UV-VIS (Jasco V-550, Tokyo, Japan) spectrophotometer. Results were expressed as gallic acid equivalent (GAE), and the quantification of the samples was performed using a calibration curve of gallic acid in concentrations varying from 0 to 1000 mg/L.

### 2.5. Determination of Carotenoid Content

Carotenoids (zeaxanthin, lutein, β-carotene, and lycopene) were extracted from the juices using liquid–liquid extraction with a mixture of hexane and acetone. More specifically, 2 mL of fruit juice, 20 mL of hexane/acetone (1:1, *v*/*v*), and 20 mL of 10% aqueous NaCl were added in a glass separatory funnel. The funnel was inverted and shaken gently for 10–20 s until two layers were formed. After separation, the aqueous phase was discarded, and the hexane layer was washed with water (20 mL) to remove acetone. The organic layer was collected, the extraction was repeated once more, and the organic phases were combined. Subsequently, sodium sulfate was added for drying, and the supernatant was transferred to a glass tube. The hexane layer was evaporated to dryness under a nitrogen flow, and the residue was dissolved in 1 mL of ethanol. The extract was filtered through a 0.45 μm PVDF filter, and a 20 μL aliquot was used for HPLC analysis.

The detection and separation of carotenoids were performed using an HPLC-DAD method. The HPLC system (Shimadzu, Kyoto, Japan) was equipped with a Degassing Unit DGU-20A5R, an LC-20AD Solvent Delivery Unit, a Sample Injector Rheodyne 7725i and an SPD-M20A Diode Array Detector. Chromatographic separation was performed in a NUCLEOSIL Macherey–Nagel C18 column (25 cm × 4.6 mm, 5 μm), and an isocratic elution was established with a mobile phase of methanol: ACN (90:10, *v*/*v*) and a flow rate of 1 mL/min. The detector was set at 450 nm for zeaxanthin, lutein, and β-carotene and at 473 nm for lycopene. The total analysis time was 30 min. Zeaxanthin and lutein are stereoisomers and could not be separated with this method; thus, they were calculated as a sum, as has been also previously reported in the literature [11,12].

### 2.6. Antioxidant Activity

The antioxidant activity (AA) of both the 4-fruit and 9-fruit juices before and after fermentation was evaluated by applying the 2,2-Azino-bis (3-ethylbenzothiazoline-6-sulfonic acid) diammonium salt (ABTS) radical cation decolorization assay [13]. ABTS•+ was prepared by reacting ABTS with potassium persulfate. Samples at five different dilutions of Trolox were analyzed within the linearity range of the assay in order to prepare the standard curve, as previously described [14]. All measurements were repeated three times. Absorbance was measured at 734 nm using a UV-VIS (Jasco V-550) spectrophotometer. The results were expressed as percent ABTS radical inhibition.

DPPH radical scavenging was determined for both juices as well using the method described by Huang et al. (2005) [14] with some minor modifications. Shortly after, 3 mL of DPPH was added to 30 μL of each sample. After incubation of the solution in the dark for 30 min, absorbance was measured at 515 nm using a UV-VIS (Jasco V-550) spectrophotometer. All measurements were repeated three times. The results were expressed as the percent of DPPH radical inhibition.

### 2.7. Sugars, Organic Acids and Ethanol Analysis

Determination of total sugars, organic acids (citric, malic, lactic, acetic, and propionic acid), and ethanol concentration was performed as previously described [15]. In brief, a Shimadzu chromatography system (Shimadzu Corp., Duisburg, Germany) equipped with a Nucleogel ION 300 OA (300 × 7.8 mm, 10 μm) column (Macherey–Nagel, Dueren, Germany), a DGU- 20A5R degassing unit, an LC-20AD pump, a CTO-20AC oven at 85 °C, and an RID-10A refractive index detector was used. H_2_SO_4_ solution (0.5 mM) was used as the mobile phase at 0.3 mL/min. Prior to analysis, 20 μL of each juice sample was filtrated with 0.22 μm filters. System calibration and data analysis were performed using LabSolutions’ integrated software (Shimadzu Corp., Kyoto, Japan). Residual sugars, ethanol, glycerol, and organic acids concentrations were calculated using standard curves prepared using reference standards (R^2^ ≥ 0.99).

### 2.8. LC-QToF/MS Analysis

A thorough profiling of phenolic compounds was performed in fermented and unfermented fruit and vegetable juices using an in-house UPLC-QtoF/MS methodology [16]. Briefly, the samples were filtered using regenerated cellulose (RC) filters and were directly injected in the analysis system comprised of an ultrahigh performance liquid chromatographic system with an HPG-3400 pump (Dionex Ultimate 3000 RSLC, Thermo Fischer Scientific, Dreieich, Germany) operating in negative ionization mode. An Acclaim RSLC C18 column (2.1 × 100 mm, 2.2 μm) from Thermo Fischer Scientific (Dreieich, Germany) was used for chromatographic separation and water/methanol (90:10 *v*/*v*, solvent A)–methanol (solvent B), both containing 5 mM of ammonium acetate, were used as the mobile phase under gradient elution conditions. All samples were analyzed in broadband collision-induced dissociation (bbCID) mode.

Target and suspect screening workflows were applied, and 29 target and suspect compounds were detected and identified, belonging to different chemical classes (phenolic acids and polyphenols). Identification of the target and suspect compounds was performed considering mass accuracy, isotopic fit, retention time, and the MS/MS fragmentation of the compounds. Moreover, for target compounds, quantification was also performed using reference standards.

### 2.9. Statistical Analysis

Bacterial counts were logarithmically transformed and presented as Log CFU/g. For comparison, unfermented 4-fruit and 9-fruit juice samples were considered as controls, and bacterial counts from the fermented juice samples using both screened strains were compared using an analysis of variance with Dunnett’s post hoc application along with a variance check at a significance level of 95%. All analyses were performed with SPSS v25 (IBM Corp, Armonk, NY, USA). Comparison of the means of each attribute at different time intervals during the sensorial evaluation was accomplished by using an analysis of variance with Tukey’s post hoc test. Minor volatiles’ concentrations (HS-SPME GC/MS) were used as variables in a principal component analysis (XLSTAT 2015.1 was used to compute the algorithm).

## 3. Results

### 3.1. Microbiological Analyses

Microbial counts in two groups of bacteria were recorded after 24 h and 48 h of fermentation for four-fruit and nine-fruit juices. The results are presented in Table 1, along with the statistical significance from the comparison (ANOVA with Dunnett’s post hoc test) of each treatment against controls.

From Table 1, it can be observed that, for the four-fruit juice, the initial number of viable cells was 8.0 ± 0.02 Log CFU/mL, and, after 24 h of fermentation, its number stabilized at 8.21 ± 0.15 Log CFU/mL and 8.44 ± 0.19 Log CFU/mL for *P. pentosaceus* SP2 and *L. paracasei* SP5, respectively. After 48 h of fermentation, cell-viable counts increased even more, up to 9.11 ± 0.23 Log CFU/mL and 9.45 ± 0.32 Log CFU/mL for *P. pentosaceus* SP2 and *L. paracasei* SP5, respectively.

In the case of the nine-fruit juice, the initial viable count was 8.4 ± 0.04 Log CFU/mL, and, after 24 h of fermentation, it climbed to 8.56 ± 0.21 Log CFU/mL and 8.41 ± 0.26 Log CFU/mL for *P. pentosaceus* SP2 and *L. paracasei* SP5, respectively. After 48 h of fermentation, the respective cell-viable counts grew rapidly to 9.35 ± 0.25 Log CFU/mL and 9.54 ± 0.11 Log CFU/mL.

### 3.2. Total Phenolic Content (TPC) Results

In order to examine the total phenolic content of the analyzed juice samples, the Folin–Ciocâlteu method was used, and the measurements are shown in Table 2.

As can be seen in Table 2, the TPCs of the unfermented four-fruit and nine-fruit juice samples were 154.34 and 143.17 mg/mL GA, respectively. The fermentation had an increasing impact on the TPC for both screened strains on both juices examined.

Specifically, after 24 h of fermentation, the application of *L. paracasei* SP5 augmented the TPC by up to 162.48 for the four-fruit juice and up to 147.23 mg/mL GA. In the case of the application of *P. pentosaceus* SP2, the respective TPC was 154.87 and 159.43 mg/mL GA for the four-fruit juice and nine-fruit juice.

Regarding the 48 h fermentation, *L. paracasei* SP5 increased TPC up to 171.30 mg/mL GA for the four-fruit juice and 150.28 mg/mL GA for the nine-fruit juice. *P. pentosaceus* SP2 exhibited the greatest TPC values, namely, 179.75 mg/mL GA when applied to the nine-fruit juice and 166.05 mg/mL GA in the case of the four-fruit juice.

### 3.3. Antioxidant Activity

**Figure 1:**
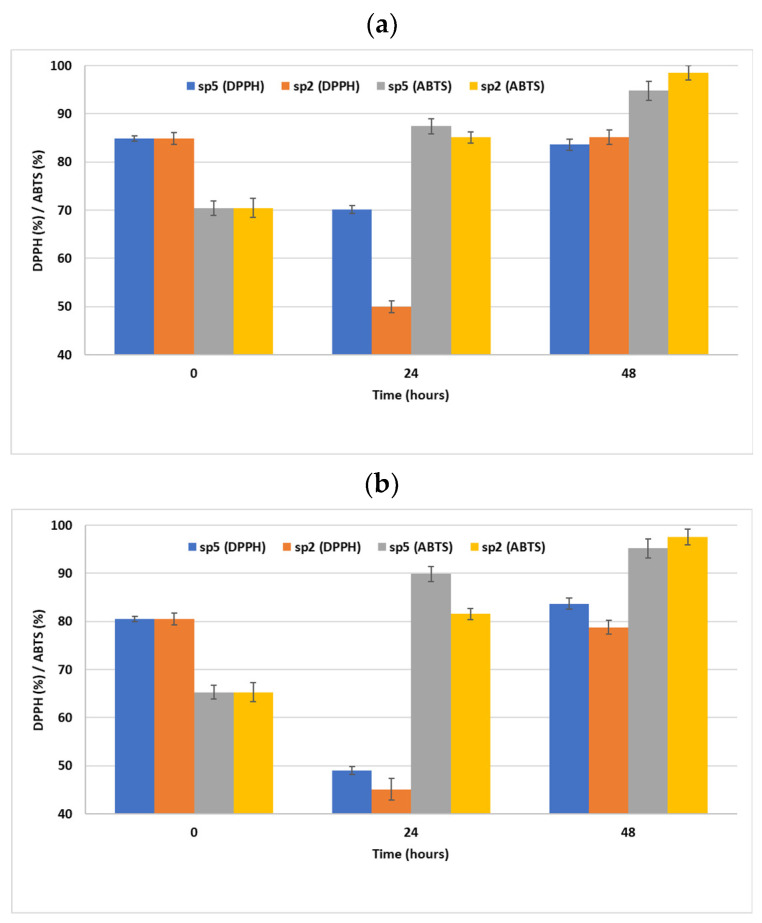
DPPH/ABTS for 4-fruit juice (**a**) and 9-fruit juice (**b**).

As can be seen in Figure 1, the DPPH radical scavenging activity of the four-fruit and nine-fruit juices was not increased by the two screened strains. On the contrary, after the 24 h fermentation of both juices using both strains, a significant decrease was recorded.

More specifically, the DPPH radical scavenging activity of the unfermented four-fruit juice sample was 84.9% and, after 24 h of fermentation, the DPPH radical scavenging activity of the samples fermented using *P. pentosaceus* SP2 and *L. paracasei* SP5 was decreased to 70.2% and 50%, respectively. After the 48 h fermentation of the four-fruit juice samples using *P. pentosaceus* SP2 and *L. paracasei* SP5, the levels of DPPH radical scavenging activity were retained as 83.61% and 85.15%, respectively. The same trend was observed in the nine-fruit juice samples, in which the DPPH radical scavenging activity was 80.53% and, after 24 h of fermentation, decreased to 49% for *L. paracasei* SP5 and to 45.1% for *P. pentosaceus* SP2.

In the case of nine-fruit juice samples, the unfermented juice exhibited an ABTS radical scavenging activity of 65.31% and, after 24 h of fermentation, the respective levels increased to 89.89% for *L. paracasei* SP5 and to 81.56% for *P. pentosaceus* SP2. The maximum ABTS radical scavenging activity was achieved after 48 h of fermentation: 95.19% for *L. paracasei* SP5 and 97.52% for *P. pentosaceus* SP2.

### 3.4. Carotenoid Content of Juice Samples

From our findings, it can be seen that the two examined strains had different behaviors during the fermentation of the two juices, as far as the carotenoids are concerned (zeaxanthin + lutein, β-carotene, and lycopene). The results are presented in Table 3.

Specifically, the unfermented four-fruit juice sample had a zeaxanthin + lutein content of 0.033 mg/L and, after 24 h of fermentation, *P. pentosaceus* SP2 increased this content to 0.091 mg/L; meanwhile, *L. paracasei* SP5 kept it stable at the same value (0.033 mg/L). After 48 h of fermentation, *P. pentosaceus* SP2 decreased the sum of zeaxanthin and lutein to 0.022 mg/L and *L. paracasei* SP5 to 0.018 mg/L. In the nine-fruit juice samples, the recorded results revealed an increase in the respective zeaxanthin + lutein content for both screened strains after both fermentation periods in comparison with the unfermented sample (0.024 mg/L). The greatest augmentation was achieved after 24 h of fermentation, namely, 0.037 mg/L for *P. pentosaceus* SP2 and 0.031 mg/L for *L. paracasei* SP5.

As for β-carotene, the unfermented four-fruit juice sample had a value equal to 0.45 mg/L, and only after 48 h of fermentation with *P. pentosaceus* SP2 was a greater content achieved (0.65 mg/L). The situation was totally differentiated in the case of the nine-fruit juice samples, which all exhibited greater values compared to the unfermented sample. *P. pentosaceus* SP2 recorded the greatest β-carotene content after 24 h of fermentation (0.83 mg/L), followed by the value for the same strain after 48 h of fermentation (0.80 mg/L) and, finally, the value for *L. paracasei* SP5 after 48 h of fermentation (0.70 mg/L).

Finally, lycopene was only detected in the four-fruit juice samples. The unfermented juice had a lycopene value of 0.007 mg/L, and this value achieved its maximum after 24 h of fermentation with *P. pentosaceus* SP2 (0.010 mg/L). The fermentation of 24 h and 48 h with *L. paracasei* SP5 provided values equal to the control (0.007 mg/L).

### 3.5. HPLC Analysis

Total sugars, organic acids (citric, acid, malic acid, lactic acid, acetic acid, and propionic acid), and ethanol content in the fermented fruit juices were monitored using HPLC and are presented in Table 4.

Organic acids constitute an important compound of food, as their concentrations may strongly affect the organoleptic attributes of food. Citric acid is, typically, one of the most abundant organic acids present in juices. However, its concentration is known to vary strongly between different fruits and vegetables [17], while fluctuations have also been noticed among different cultivars as well as between different maturity stages [18]. In our work, both commercial juices (containing different fruit and vegetable mixtures) presented relatively high initial citric contents (derived mainly from citrus fruits), which were easily metabolized by both microorganisms, especially after 48 h. Similarly, malic acid content demonstrated a significant decline through fermentations (for both juices) and was accompanied by lactic acid production in all cases (up to 4.1 ± 0.3 g/L) as a by-product of malolactic fermentation. Notably, no initial levels of lactic acid were present in the fruit juices. Acetic acid was detected in both fermented fruit juices only when *P. pentosaceus* SP2 was used but still in very low concentrations (≤0.3 ± 0.1 g/L). On the other hand, propionic acid was found only in the fermented four-fruit mixed juices in very small amounts (<0.5 ± 0.1 g/L).

Despite the microbial activity, total sugar levels appear to have remained stable or only slightly diminished during the first 24 h of fermentation in all cases. This may seem uncommon; however, in other works, similar variations, or even sugar content rises, were noticed probably due to polysaccharides hydrolysis through the action of pectinases or other enzymes [19,20,21]. After 48 h of fermentation, however, total sugar concentrations decreased significantly in all cases, and small amounts of ethanol were detected (ranging from 0.3 to 0.7 ± 0.1%), as previously noted in the lactic acid fermentation of pomegranate juice [10]. Nevertheless, the possibility of a strain demonstrating heterofermentative action cannot be excluded.

The pH values of the four-fruit juice fermented using *P. pentosaceus* SP2 revealed a minor variation after 48 h of fermentation (pH value of 3.27) in comparison to the unfermented juice samples (pH value of 3.34). Moreover, for *L. paracasei* SP5, after 48 h of fermentation, the pH value dropped to 3.18. In the case of the nine-fruit juice, *P. pentosaceus* SP2 and *L. paracasei* SP5 decreased the pH values to 3.38 and 3.37, respectively, after 48 h of fermentation compared to the unfermented sample, which exhibited a pH value of 3.59.

### 3.6. LC-QToF/MS Phenolic Profiling

Twentynine phenolic compounds were detected and identified in the four-fruit and nine-fruit juices before and after fermentation. Bearing in mind the results from the calculation of TPC and antioxidant activity, it was important to obtain a clearer picture of the bioactive compounds that might enhance the antioxidant profile of the juices examined. Following the target screening workflow, 19 compounds were detected and quantified, including seven phenolic acids (abscisic acid, salicylic acid, chlorogenic acid, gallic acid, quinic acid, protocatechuic acid, gentisic acid), four phenols (catechin, epicatechin, phloridzin, polydatin), two phenyl alcohols (tyrosol, hydroxytyrosol), two ethyl esters of phenolic acids (ethyl caffeate, ethyl gallate), one flavanone (hesperetin), one flavonoid (naringenin), and two flavonols (quercetin, rutin) (Table 5). Moreover, following a suspect screening workflow, we were able to identify 10 more phenolic compounds, often occurring in fruit juices but with no reference standards available. These compounds were hesperidin, limonin, procyanidins B1 and B2, vicenin-2, eriocitrin, caffeoylquinic acid, and three coumaroylquinic acid isomers. Suspect compounds could not be quantified due to the lack of reference standards; however, the impact of the probiotic strains on their content was assessed using the peak area of the chromatographic peaks. The results of the suspect screening of fruit juices are presented in Supplementary Materials (Figure S1).

The impact of *P. pentosaceus* SP2 and *L. paracasei* SP5 was not the same for the four-fruit and nine-fruit juices, most probably due to the different fruits included. More specifically, in the four-fruit juice, *L. paracasei* SP5 increased 14—and *P. pentosaceus* SP2 18—out of the 29 phenolic compounds determined; meanwhile, in nine-fruit juice, *L. paracasei* SP5 increased 17 and *P. pentosaceus* SP2 15.

As far as the four-fruit juice is concerned, a significant augmentation was observed to quinic acid, with the juice fermented with *P. pentosaceus* SP2 exhibiting a concentration of 21 mg/L and the juice fermented with *L. paracasei* SP5 exhibiting a concentration of 16.3 mg/L. This corresponds to an increase of 110% and 60%, respectively, compared to the 10 mg/L quinic acid calculated in the control. An even higher enhancement of tyrosol was observed after 48 h of fermentation, with both the probiotic strains reaching 3530% for *P. pentosaceus* SP2 (control sample 0.14 mg/L, fermented sample 5.08 mg/L) and 1680% for *L. paracasei* SP5 (control sample 0.14 mg/L, fermented sample 2.49 mg/L). The fermentation with *P. pentosaceus* SP2 was also found to have a significant impact on gallic acid (55% increase) and protocatechuic acid (50% increase), compounds that did not exhibit the same behavior when *L. paracasei* SP5 was used. On the contrary, the fermentation with *L. paracasei* SP5 resulted in a significant increase in ethyl gallate (71% higher concentration than the control). Caffeoylquinic acid and coumaroylquinic acid isomers were also found to be elevated.

As for the nine-fruit juice, similar results were obtained with some differences. Again, quinic acid and tyrosol presented an augmentation of their concentrations; however, this augmentation was notably smaller than for the four-fruit juice. 2-cis,4-trans-abscisic acid, ethyl gallate, and gallic acid were increased with both strains examined; meanwhile, from suspect screening results, it was observed that vicenin-2, procyanidins B1 and B2, and caffeoylquinic acid demonstrated an enhancement when *P. pentosaceus* SP2 was used compared to *L. paracasei* SP5, while eriocitrin was slightly increased with the use of *L. paracasei* SP5.

A notable decrease was recorded in the levels of chlorogenic acid in all the fermentations conducted. As for the four-fruit juice, the control exhibited 19 mg/L, *P. pentosaceus* SP2: 14 mg/L and *L. paracasei* SP5:12 mg/L; moreover, as for the nine-fruit juice, the control scored 27 mg/L, with both *P. pentosaceus* SP2 and *L. paracasei* SP5 reaching 21 mg/L.

## 4. Discussion

From the results of bacterial growth, it can be concluded that both strains achieved a greater rate of increase after 48 h of fermentation in both juices. Nevertheless, *L. paracasei* SP5 exhibited the highest viable cell counts after 48 h of fermentation (9.45 Log CFU/mL and 9.54 Log CFU/mL, respectively). Our findings are in accordance with those of Yang et al. (2022) [22], where three LAB strains, namely, *L. acidophilus, L. casei*, and *L. plantarum*, showed a cell viability of approximately 8.5 Log CFU/mL after 48 h of fermentation in apple juice. Similarly, in a previous work conducted in our laboratory, the viability of *L. paracasei* SP3 after 24 h of fermentation was 9.2 Log CFU/mL in pomegranate juice [23]. Bioactive compounds in juices could function as prebiotics for the survival of probiotics [24] This phenomenon may explain the increased survivability in both fermented juice samples.

It is known that LAB strains can metabolize sugar and produce organic acids, thus changing the pH value of the respective juice during fermentation. These variations in pH values are in line with other researchers’ findings, which showed similar pH decreases when LAB were applied to ferment fruit juices [25].

As for the total phenolic content, it is well established in the literature that total phenolic content is influenced by several factors, such as fruit variety, processing methods, and storage conditions [26]. Moreover, processing methods during juice production, like clarification and filtration, might remove part of the phenolic compounds that bound to the fiber and pectin [27], and heat treatment might degenerate anthocyanins found in large amounts in grapes [28]. It is known that high levels of punicalagin are found in some juices, especially those containing pomegranate [29]. The fact that phenolic compounds are the main contributors to antioxidant activity in terms of radical scavenging cannot entirely predict the DPPH or/and ABTS percentage since vitamin C and carotenoids also partially contribute to antioxidant functions [30]. Wern et al. (2016) [31] recorded total phenolic content values in commercial 100% fruit juices, reaching 1.02 mg/100 mL in the case of grape and 1.30 mg/100 mL in the case of pomegranate.

Previous studies have also shown that plant parts exhibit an increase in phenolic content after fermentation [32]. Our results demonstrated a significant increase after 24 h and 48 h of fermentation as well. The capacity of LAB to produce hydrolytic enzymes, which decompose complex polyphenols into simpler flavonols during fermentation, may explain the enhanced TPC in the fermented four-fruit and nine-fruit juice samples. Our results are in accordance with Kwaw et al. (2018) [33], who reported that, during fermentation with *L. plantarum* ATCC SD 5209, the TPC of fermented mulberry juice was enhanced. Similarly, lactic acid fermentation of kiwi fruit juice [34], ginkgo kernel juice [35], and blueberry juice [1] provided increased TPC in the fermented juice samples. Our total phenolic content values ranged from 1.43 and 1.54 mg/100 mL (before fermentation) to 1.79 and 1.71 mg/100 mL (after fermentation). Similar findings have been recorded by Yang X et al. (2018) [36], with total phenolic content reaching 12.1 mg/100 mL after the eighth day of fermentation of mixed juices containing apples, pears, and carrots with two commercial *L. plantarum* strains. The differences in the TPC among the LAB strains have been attributed to the ability of the strains to produce more hydrolytic enzymes in different matrices [37].

Furthermore, the carotenoid content and zeaxanthin+ lutein content increased in both juices by both screened strains after fermentation, with the only exception being the case of the four-fruit juice with *P. pentosaceus* SP2 after 48 h of fermentation. β-carotene content increased after 48 h of fermentation for both strains, whereas the four-fruit juice samples decreased, except in the case of *P. pentosaceus* SP2 after 48 h of fermentation, where an increase in β-carotene content was recorded. Similarly, in another work, β-carotene, trans-β-carotene, zeaxanthin, lutein, and α-carotene levels increased significantly in all fermented mango juice samples compared to their respective unfermented mango juice samples with higher concentrations in *Leuconostoc pseudomesenteroides* fermented ‘Sabre’ mango juice and a significant 2.59-fold increase [37].

The observed increase during fermentation could be explained by two factors: (i) the release of carotenoids as an immediate response to macromolecular changes in the juice during fermentation and (ii) the increased synthesis of carotenoids by LAB during the fermentation process since LAB are known to synthesize carotenoids as a protective mechanism against oxidative stress [38]. Recently, Tian et al. (2020) [39] recorded that LAB fermentation can also enhance the release and bioaccessibility of phytochemicals, such as carotenoids, in plant matrix.

Regarding HPLC analysis, several organic acids were produced and identified, followed by a decrease in total sugars. The main organic acid was lactic acid, and it was produced in respectable amounts after 24 and 48 h of fermentation, while the low alcohol content (<1% *v*/*v*) met the standards set for low or non-alcoholic beverages [40]. At the same time, a small decrease in malic acid levels was recorded, thus demonstrating a low rate of malolactic fermentation. This phenomenon is not strange, considering that it is possible for the applied LAB, due to adaptation conditions at the beginning of concentration, to select a carbon source from malic acid in order to begin the fermentation [41]. Propionic and acetic acids were produced in low concentrations in most cases, probably due to the enzymatic degradation of the citric acid naturally present in fruit juice, which is verified through analysis [42]. In particular, levels of citric acid were decreased in all cases after 24 and 48 h of fermentation. The pH value of the fermented juices was monitored in all of the studied periods. No significant alteration was recorded due to the high buffering capacity of the fermented juices, as other researchers have reported in similar experiments [42].

As far as antioxidant activity is concerned, our results indicated an overall increase after 48 h of fermentation in both juice substrates using both screened strains. Nevertheless, after 24 h of fermentation with the DPPH method, a decrease was recorded for both strains and both juices examined. Other works have shown similar results, indicating a rise in DPPH radical scavenging capacity in blueberry juice, suggesting that LAB fermentation has a positive impact on the phenolic compounds for proton donation [43]. Thus, DPPH radical scavenging capacity and TPC positively correlate between them. A positive correlation also exists between the DPPH radical scavenging capacity and some phenolic acids (quinic acid, protocatechuic acid), some flavonoids (rutin, naringenin), and catechin and epicatechin, which are effective DPPH radical scavengers [34].

Similar findings were recorded by Wang K et al. (2022) [44] after the fermentation of mulberry juice. The observed increase in the DPPH radical scavenging capacity of the fermented juice samples might be attributed to the rapid degradation of anthocyanins in order to produce intermediate metabolites with low antioxidant activity in the early fermentation stage. After that, they entirely transformed into phenolics with high antioxidants after fermentation [25]. Furthermore, LAB fermentation induced a rise in the ABTS radical scavenging capacity, which proved that LAB could promote the transfer of electrons to scavenge ABTS free radicals in both the fermented four-fruit and nine-fruit juice samples. Our findings are in line with Jia et al. (2022) [45], who assumed that the resonance between the aromatic benzene rings of the phenolic hydroxyl groups and the free electron pair on the phenolic oxygen can accelerate the delocalization of the electrons, increasing resistance from the oxygen free radicals.

As far as phenolic compounds are concerned, it has been established that the LAB biotransformation of phenolics is matrix- and LAB strain-dependent in mango juice samples [37] and orange juice samples [46]. Moreover, LAB are able to synthesize a series of enzymes (benzyl–alcohol–dehydrogenases, decarboxylases, tannases) in order to degrade phenolics [42].

More specifically, the content of catechin in apple juice fermented by *L. acidophilus*, *L. plantarum*, and *L. casei* increased while the content of protocatechuic acid was decreased, after fermentation. In the same work, it was reported that LAB can metabolize protocatechuic acid to catechin through the decarboxylation reaction [47]. Our results indicated that protocatechuic acid was increased after 48 h of fermentation with *P. pentosaceus* SP2 and not detected after 48 h of fermentation with *L. paracasei* SP5 in the case of the four-fruit juice samples. In the nine-fruit juice samples, the levels of protocatechuic acid remained stable after 48 h of fermentation for both strains. The study by He et al. (2011) [48] also demonstrated the metabolic correlation between protocatechuic acid and catechin. The loss of phenolics in apple juice has been attributed to the interaction between phenolics and proteins during fermentation, which could produce insoluble complexes [49].

Additionally, rutin and quercetin could be biotransformed to low-molecular-weight metabolites [50]. In our work, rutin and quercetin were detected only in nine-fruit juice samples; moreover, rutin increased its content and quercetin decreased its content after 48 h of fermentation using both screened strains. Our results are in accordance with Li et al. (2021) [51], who reported that LAB fermentation increased the rutin content in the jujube juice.

Moreover, Kwaw et al. (2018) [33] recorded that, in mulberry juice, the content of rutin and epicatechin was upregulated after LAB fermentation. In our results, epicatechin content was increased only in the case of *P. pentosaceus* SP2 in both juice samples examined. Tao et al. (2022) [52] exhibited that, after 48 h of fermentation, epicatechin in “Fuji” apple juice using LAB strains was metabolized to form gallic acid and phloretin. Gallic acid content was increased in our findings as well.

Additionally, chlorogenic acid can be metabolized into other phenolic compounds using the LAB strains [34]. This fact could explain the decrease in chlorogenic acid content in our work as well.

As for tyrosol, it is known to be a biologically active phenolic compound in fruit juices and wine with anti-inflammatory effects that prevent cardiovascular diseases and melanin pigmentation [53]. The metabolic pathways of tyrosol biosynthesis include the derivation of phenylalanine and tyrosine. The former pathway includes the conversion of phenylalanine to cinnamic acid, followed by the conversion to p-coumaric acid and, subsequently, to tyrosol, and the latter includes the conversion of tyrosine to tyramine and, finally, into tyrosol [54].

Furthermore, the recorded increase in quinic acid may be attributed to the hydrolysis of chlorogenic acid caused by the two LAB strains, which leads to the synthesis of caffeic acid and quinic acid. A reduction in the respective concentration of chlorogenic acid was observed in both LAB strains and in both fruit juices after fermentation. Similar findings were recorded by Filannino et al. (2014) [55] after the application of *L. reuteri* FUA3168 into cherry juice. Quinic acid may be further metabolized to catechol using *Lactobacillus* spp. Nevertheless, catechol was not detected in our results.

Moreover, other compounds existing in fruit juices may exhibit prebiotic properties and promote the growth of LAB. In many in vitro studies, the promotion of the levels of Bifidobacteria and Lactobacilli has been reported to increase after the lactic acid fermentation of grape seed extract due to polyphenols anthocyanins [49]. In particular, it has been reported that, probably, anthocyanins are transformed into small molecular phenolic acids either by gut microbiota or by an LAB strain through fermentation and various reactions. The metabolites produced from polyphenols seem to have prebiotic effects stimulating the growth of beneficial bacteria and obstructing the propagation of harmful bacteria [42]. However, these metabolic reactions concerning the catabolism of anthocyanins are numerous and difficult to explain in depth and need more research with in vitro and even in vivo tests.

As a bottom line, it can be concluded that the nutritional value of both fermented mixed fruit juices was ameliorated in terms of phenolic compounds, antioxidant activity, total phenolic content, carotenoid content, and probiotic viability.

## 5. Conclusions

Potential probiotics *L. paracasei* SP5 and *P. pentosaceus* SP2 were applied successfully in the lactic acid fermentations of four-fruit (red apple, orange, pomegranate, and red grape) and nine-fruit (red apple, red grape, cherry, pomegranate, sour cherry, strawberry, black chokeberry, blueberry, blood orange, and black carrot) mixed juices. Respectable amounts of lactic acid were produced, while the viability of both strains was preserved at respectable levels (over 8 Log CFU/mL) in all the studied periods. Nutritional enhancements were recorded in all fermented juices in terms of carotenoid content, the composition of the phenolic compounds, as well as the TPC and AA levels. Nevertheless, both LAB strains were efficient in lactic acid fermentation and their technological properties.

## Figures and Tables

**Table 1:** Bacterial counts (mean Log CFU/mL ± SD of three samples) of the fermented juice samples after 24 h and 48 h of fermentation.

Fruit Juice	Strains	Time	LAB	Yeasts/Fungi
			Log CFU/mL	
		0 h	8.0 ± 0.0	
4 FRUITS	*P. pentosaceus* SP2	24 h	8.21 ^a^ ± 0.15	nd
		48 h	9.11 ^b^ ± 0.23	nd
	*L. paracasei* SP5	24 h	8.44 ^a^ ± 0.19	nd
		48 h	9.45 ^b^ ± 0.32	nd
		0 h	8.4 ± 0.0	
9 FRUITS	*P. pentosaceus* SP2	24 h	8.56 ^a^ ± 0.21	nd
		48 h	9.35 ^b^ ± 0.25	nd
	*L. paracasei* SP5	24 h	8.41 ^a^ ± 0.26	nd
		48 h	9.54 ^b^ ± 0.11	nd

nd: not detected (no visible colony or less than 10 cfu/mL). ^a,b^ Different superscript letters in columns for the same fruit juice indicate statistically significant differences (multifactor (MF)-ANOVA with Tukey’s honestly significant difference (HSD) multiple range test).

**Table 2:** Total phenolic content of the juice samples, expressed as mg/mL of gallic acid.

Fruit Juice	Strains	Time	TPC
			mg/mL GA
4 FRUITS		0 h	154.34 ^a^ ± 4.21
	*P. pentosaceus* SP2	24 h	164.87 ^b^ ± 3.85
		48 h	166.05 ^b^ ± 3.41
	*L. paracasei* SP5	24 h	162.48 ^b^ ± 4.10
		48 h	171.30 ^b^ ± 2.90
9 FRUITS		0 h	143.17 ^a^ ± 3.42
	*P. pentosaceus* SP2	24 h	159.43 ^b^ ± 4.19
		48 h	179.75 ^c^ ± 5.28
	*L. paracasei* SP5	24 h	147.23 ^a^ ± 3.16
		48 h	150.28 ^a^ ± 4.31

^a–c^ Different superscript letters in columns for the same fruit juice indicate statistically significant differences (multifactor (MF)-ANOVA with Tukey’s honestly significant difference (HSD) multiple range test).

**Table 3:** Carotenoid content of 4-fruit and 9-fruit juice samples after 24 h and 48 h of fermentation.

Fruit Juice	Strains	Time	Zeaxanthin + Lutein	β-Carotene	Lycopene
			C (mg/L)		
4 FRUITS		0 h	0.033	0.45	0.007
	*P. pentosaceus* SP2	24 h	0.091	0.42	0.010
		48 h	0.022	0.65	0.006
	*L. paracasei* SP5	24 h	0.033	0.37	0.007
		48 h	0.018	0.18	0.007
9 FRUITS		0 h	0.024	0.44	nd
	*P. pentosaceus* SP2	24 h	0.037	0.83	nd
		48 h	0.036	0.80	nd
	*L. paracasei* SP5	24 h	0.031	0.48	nd
		48 h	0.026	0.70	nd

**Table 4:** HPLC analysis and major volatiles detected in juice samples.

Fruit Juice	Strains	Time	Citric Acid	Malic Acid	Lactic Acid	Acetic Acid	Propionic Acid	Total Sugars	Ethanol	pH
			g/L						%vol	
4 fruits		0 h	5.1 ± 0.1	4.1 ± 0.1	nd	nd	nd	118.9 ± 1.1	nd	3.34
	*P. pentosaceus* SP2	24 h	4.6 ± 0.2 ^a^	1.7 ± 0.2 ^a^	2.9 ± 0.2 ^a^	0.1 ± 0.1 ^a^	nd	112.1 ± 0.6 ^a^	nd	3.37
		48 h	4.3 ± 0.2 ^a^	0.9 ± 0.1 ^b^	3.3 ± 0.2 ^a^	0.1 ± 0.1 ^a^	nd	84.1 ± 0.8 ^b^	0.5 ± 0.1	3.27
	*L. paracasei* SP5	24 h	4.7 ± 0.1 ^a^	4.1 ± 0.2 ^a^	1.3 ± 0.1 ^a^	nd	nd	114.6 ± 0.4 ^a^	nd	3.28
		48 h	4.2 ± 0.3 ^b^	3.7 ± 0.2 ^a^	1.2 ± 0.1 ^a^	nd	nd	85.3 ± 2.1 ^b^	0.5 ± 0.1	3.18
9 fruits		0 h	4.3 ± 0.1	2.9 ± 0.1	nd	nd	nd	116.5 ± 0.8	nd	3.59
	*P. pentosaceus* SP2	24 h	4.0 ± 0.1 ^a^	2.2 ± 0.1 ^a^	3.5 ± 0.2 ^a^	0.3 ± 0.1 ^a^	0.5 ± 0.1 ^a^	114.6 ± 0.3 ^a^	nd	3.55
		48 h	3.0 ± 0.3 ^b^	2.0 ± 0.1 ^a^	4.1 ± 0.3 ^b^	0.1 ± 0.1 ^b^	0.5 ± 0.1 ^a^	84.5 ± 1.9 ^b^	0.7 ± 0.1	3.51
	*L. paracasei* SP5	24 h	4.2 ± 0.1 ^a^	2.3 ± 0.1 ^a^	2.8 ± 0.1 ^a^	nd	0.5 ± 0.1 ^a^	116.7 ± 0.4 ^a^	nd	3.38
		48 h	2.8 ± 0.2 ^b^	2.1 ± 0.1 ^a^	2.7 ± 0.2 ^a^	nd	0.5 ± 0.1 ^a^	83.3 ± 1.3 ^b^	0.3 ± 0.1	3.37

^a,b^ Different superscript letters in columns indicate statistically significant differences (ANOVA, Duncan Post Hoc Multiple Comparisons), nd: not detected (<0.1 g/L).

**Table 5:** Target phenolic compounds detected and quantified in fermented (48 h) and unfermented juice samples in mg/L, using LC-QTοF/MS.

	4 FRUITS			9 FRUITS		
	Control	*P. pentosaceus* SP2	*L. paracasei* SP5	Control	*P. pentosaceus* SP2	*L. paracasei* SP5
Target Compounds	Concentration (mg/L)					
2-cis,4-trans-Abscisic acid	0.12	0.12	0.14	0.069	0.085	0.083
Salicylic acid	0.094	0.083	0.082	0.16	0.12	0.12
Catechin	0.45	0.44	0.36	0.61	0.68	0.66
Epicatechin	0.70	0.85	0.67	1.2	1.2	1.2
Chlorogenic acid	18	14	12	27	21	21
Ethyl caffeate	0.022	0.030	0.027	ND	ND	ND
Ethyl gallate	0.14	0.16	0.24	0.081	0.11	0.10
Gallic acid	1.1	1.7	1.1	0.43	0.53	0.51
Hesperetin	ND	ND	ND	0.091	0.073	0.094
Hydroxytyrosol	0.14	0.13	0.13	0.074	0.072	0.063
Phloridzin	4.7	5.6	4.8	1.8	1.5	1.4
Polydatin	0.12	0.11	0.063	0.21	0.092	0.22
Quercetin	ND	ND	ND	0.22	0.14	0.17
Quinic acid	10	22	16	16	23	19
Rutin	ND	ND	ND	0.86	1.0	0.95
Tyrosol	0.14	5.1	2.5	0.15	2.5	1.2
Naringenin	ND	ND	ND	0.046	0.045	0.049
3,4-Dihydroxybenzoic acid (Protocatechuic acid)	1.0	1.5	ND	1.1	1.1	1.0
2,5-Dihydroxybenzoic acid (Gentisic acid)	0.10	0.17	0.092	0.31	0.31	0.22

## Data Availability

The original contributions presented in the study are included in the article/Supplementary Materials, further inquiries can be directed to the corresponding author.

## Supplementary Materials

The following supporting information can be downloaded at: https://www.mdpi.com/article/10.3390/foods13071136/s1, Figure S1: Variation of suspect phenolic compounds in juices before and after fermentation with *P. pentosaceus* SP2 and *L. paracasei* SP5.
