# Supplementary material for: Chemical Profile Characterization of Fruit and Vegetable Juices after Fermentation with Probiotic Strains
Source: Foods. 2024 Apr 8;13(7):1136. doi: 10.3390/foods13071136 (PMC11011985; doi:10.3390/foods13071136)
Supplement: Supplementary file 1 [file foods-13-01136-s001.zip › foods-2925637-supplementary.pdf]

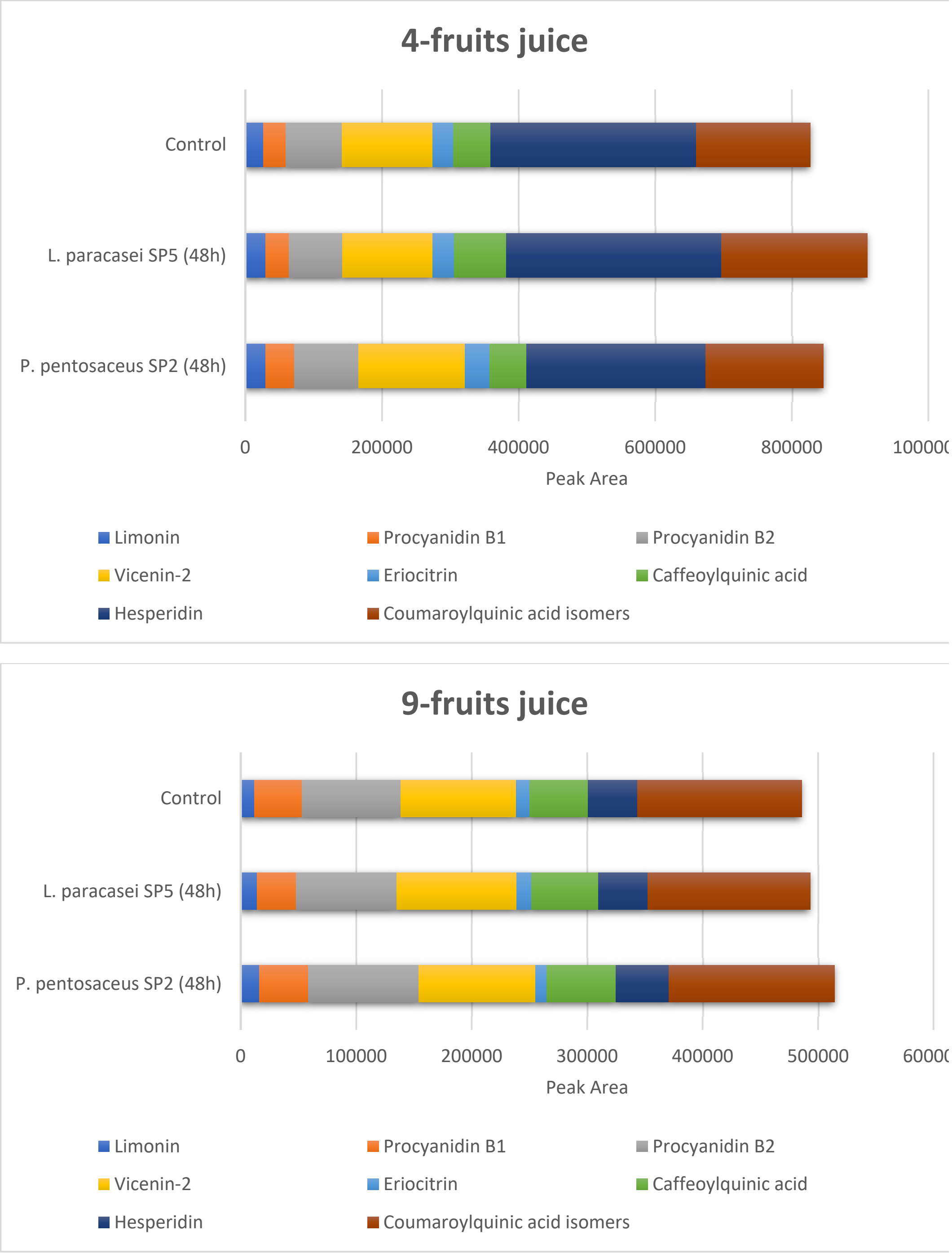

Figure S1. Variation of suspect phenolic compounds in juices before and after fermentation with *P. pentosaceus* SP2 and *L. paracasei* SP5.
